# Supplementary material for: Content-rich biological network constructed by mining PubMed abstracts
Source: BMC Bioinformatics. 2004 Oct 8;5:147. doi: 10.1186/1471-2105-5-147 (PMC528731; doi:10.1186/1471-2105-5-147)
Supplement: Additional File 5 — The original Chilibot query results of the term "long-term potentiation (LTP)" and 22 other terms, limiting the latest references analyzed to the years 1990, 1995, 2000, and 2004. [file 1471-2105-5-147-S5.bz2 › chilibotAdditionalFile5/ltp2000/html/PKC_PKA.html]

 


 **PKC** and **PKA** 
  
Found 1035 abstracts in PubMed,  **30 abstracts were retrieved and analyzed**.  


---

 Search Google  |
 PDF files only 
|  EDU domain only 

---

**Interactive relationship** (e.g. stimulation, inhibition, etc)

- Induction of c Fos and stimulation of DNA synthesis by ACTH are independent of  **PKA**  and are inhibited by the  **PKC**  inhibitor GF109203X.  Ref: 11004713 Braz J Med Biol Res, 2000
- Dexamethasone further enhanced FSK or PMA induced proENK mRNA expression, which was not correlated with the activation of AP 1 expression and CREB phosphorylation, suggesting that synergistic interaction of glucocorticoid with  **PKA**  or  **PKC**  pathway for the regulation of proENK mRNA expression appears to be mediated by other pathways rather than CREB and AP 1 families.  Ref: 11113530 Brain Res Mol Brain Res, 2000
- Therefore, for the potentiation of transfection by the TPA TFL 01 method, conventional  **PKC**  activity with significant but low protein kinase A  **PKA**  activity are first required, and then a novel  **PKC**  activity with significant  **PKA**  activity.  Ref: 11098142 J Biochem (Tokyo), 2000
- Because  **PKC**  activity regulates N cadherin expression and chondrogenesis, the  **PKA**  mediated regulation of PKCalpha appears to be responsible for the  **PKA**  regulation of N cadherin expression and chondrogenesis.  Ref: 11092400 J Bone Miner Res, 2000

**Parallel relationship** (e.g. studied together, co-existance, homology, etc.)

- Here we show that the human PER1 hPER1 gene is synergistically activated by protein kinases A and C  **PKA** ,  **PKC**  and cAMP responsive element binding protein.  Ref: 11119726 FEBS Lett, 2000
- The association between the phosphorylation status of the retinoblastoma protein, pRb and changes in cell cycle control caused by either protein kinase C  **PKC**  or protein kinase A  **PKA**  stimulation was evaluated in human myeloblastic leukaemia ML 1 cells.  Ref: 11041200 Cell Prolif, 2000
- Activators and inhibitors of  **PKA**  as well as  **PKC**  modulate endogenous hPER1 expression and hPER1 promoter driven reporter gene activity in a dose dependent manner.  Ref: 11119726 FEBS Lett, 2000
- The effect on BAC was time dependent, and mechanistically did not appear to be the result of inhibition of protein kinase C  **PKC** , protein kinase A  **PKA**  or MEK 1.  Ref: 11069728 Osteoarthritis Cartilage, 2000
- TS 2 and TS 4 showed much weaker inhibitory activity toward  **PKA**  and  **PKC** , while TS 4... moderately inhibited immunoprecipitated v src kinase.  Ref: 10913818 Biochim Biophys Acta, 2000
- These studies demonstrate that the molecular mechanism of NE action on the expression of the ANG gene in OK cells may be mediated via both the  **PKA**  and  **PKC**  signalling pathways and via the phosphorylation of CREB.  Ref: 11108138 Mol Cell Biochem, 2000
- Effects of HCO 3 on protein kinase C  **PKC**  and protein kinase A  **PKA**  induced anion conductances were investigated in Necturus gallbladder epithelial cells.  Ref: 11029286 Am J Physiol Cell Physiol, 2000
- The AR is regulated by phosphorylation and cross talk with several signaling pathways, including MAP kinases,  **PKA**  and  **PKC** .  Ref: 11127964 Prog Drug Res, 2000
- Phosphorylation consensus sites for various kinases exist within the sequence of the potassium channel subunit Kv4.2, including consensus sites for extracellular signal regulated kinase mitogen activated protein kinase ERK MAPK, protein kinase A  **PKA** , protein kinase C  **PKC** , and calcium calmodulin dependent kinase II CaMKII, and kinase assays have shown that particular amino acids of the consensus sites are bonafide phosphorylation sites in vitro.  Ref: 11040264 Learn MemLearn Mem, 2000
- We conclude that the long and short forms of the 5 HT 3 receptor are differentially modulated by agents that activate  **PKA**  and  **PKC** .  Ref: 11000482 Brain Res Mol Brain Res, 2000
- In HEK 293 cells, PGI 2, acting through endogenous AH6809 sensitive EP 1 rather than IP receptors, and the selective EP 1 receptor agonist 17 phenyl trinor PGE 2 antagonized U46619 mediated signalling by both TPalpha and TPbeta receptors in a  **PKC**  dependent,  **PKA**  independent manner.  Ref: 11015313 Br J Pharmacol, 2000
- The results in this paper indicate that LPS induced NF kappa B binding activity was independent of  **PKC** ,  **PKA** , ERK, and p38 MAPK, but was regulated by proteasome.  Ref: 11006016 Cell Immunol, 2000
- In a multiple protein kinase assay using a postnuclear fraction of v src transformed NIH3T3 cells, 4 ethyl 4 hydroxy 2 p tolyl dihydro 4H selenazine TS 2 and 4 hydroxy 6 isopropyl 4 methyl 2 p tolyl dihydro 4H selenazine TS 4 exhibited selective inhibitory activity against eukaryotic elongation factor 2 kinase eEF 2K over protein kinase A  **PKA** , protein kinase C  **PKC**  and protein tyrosine kinase PTK.  Ref: 10913818 Biochim Biophys Acta, 2000
- Therefore, CPO activates the ERK 44 42 signaling cascade in CHOK1 cells via a pathway dependent on P13 K,  **PKC** , and MEK but not requiring  **PKA**  or the human M2 muscarinic receptor.  Ref: 11083088 J Biochem Mol Toxicol, 2000
- Accordingly, this study aimed at resolving possible involvement of cAMP  **PKA**  and diacylglycerol  **PKC**  in the conopressin response.  Ref: 11067996 J Neurophysiol, 2000
- ... the blockade of  **PKC** ,  **PKA** , and ERK had little effect.  Ref: 11006016 Cell Immunol, 2000
- HG resulted in a time dependent increase in total  **PKC**  and  **PKA**  activities, 57 11.3 and 85 17.4%, respectively.  Ref: 10997914 Am J Physiol Renal Physiol, 2000
- Furthermore, our data suggest that the effects of hCG and GnRH on ERalpha and ERbeta expression in hGLCs are mediated in part by activation of  **PKA**  and  **PKC**  signaling pathways, respectively.  Ref: 11061546 J Clin Endocrinol Metab, 2000
- Sustained activation of the conductance by  **PKA**  was observed in calcium free conditions 5 mM BAPTA in the electrode solution, or when 100 nM calphostin C, a  **PKC**  inhibitor, was added to the electrode solution.  Ref: 11041561 Pflugers Arch, 2000
- Pharmacological downregulation of  **PKC**  by long term incubation of MES cells with 0.5 microM phorbol 12 myristate 13 acetate or inhibition of  **PKA**  activity by 2 microM H 8 blocked the effects of HG and GlcN on laminin synthesis.  Ref: 10997914 Am J Physiol Renal Physiol, 2000
- this stimulation involves  **PKC**  and  **PKA**  signaling pathways.  Ref: 10997914 Am J Physiol Renal Physiol, 2000
- These results suggest that a dynamic shift in the binding of TF to TRE from  **PKA**  to  **PKC**  responsive TF occurs during palate development.  Ref: 11097866 Toxicol Appl Pharmacol, 2000
- In summary, exocytosis in epithelial cells can be stimulated directly by calcium,  **PKA** , or  **PKC** , and is mediated by physical mechanisms similar to those in neurons and endocrine cells.  Ref: 11004201 J Gen Physiol, 2000
- The effect of ET 1 on glucose uptake was not inhibited by inhibitors for protein kinase C  **PKC** , protein kinase A  **PKA**  and phosphatidylinositol 3 kinase PI3 kinase.  Ref: 11078371 J Cardiovasc Pharmacol, 2000
- A specific protein kinase A  **PKA**  inhibitor, H 89, but not the  **PKC**  inhibitor K252a reversed the down regulation of 1 5 EET receptor binding caused by dibutyryl cAMP in GPM cells.  Ref: 11060896 Prostaglandins Other Lipid Mediat, 2000
- We suggest that neither  **PKC**  nor  **PKA**  is required to induce LTP at this synapse.  Ref: 11069602 Eur J Neurosci, 2000
